# A novel *PIK3R1* mutation of SHORT syndrome in a Chinese female with diffuse thyroid disease: a case report and review of literature

**DOI:** 10.1186/s12881-020-01146-3

**Published:** 2020-10-31

**Authors:** Liying Sun, Qianwen Zhang, Qun Li, Yijun Tang, Yirou Wang, Xin Li, Niu Li, Jian Wang, Xiumin Wang

**Affiliations:** 1 Department of Pediatric and Adolescent Gynecology, The Children’s Hospital of Zhejiang University School of Medicine, National Clinical Research Center for Child Health, Hangzhou, China; 2 Department of Endocrinology and Metabolism, Shanghai Children’s Medical Center, Shanghai Jiao Tong University School of Medicine, Shanghai, China; 3 Department of Medical Genetics and Molecular Diagnostic Laboratory, Shanghai Children’s Medical Center, Shanghai Jiao Tong University School of Medicine, Shanghai, China

**Keywords:** SHORT syndrome, *PIK3R1* gene, Whole-exome sequencing, Novel variant, Case report

## Abstract

**Background:**

SHORT syndrome is a rare genetic disease named with the acronyms of short stature, hyper-extensibility of joints, ocular depression, Rieger anomaly and teething delay. It is inherited in an autosomal dominant manner confirmed by the identification of heterozygous mutations in *PIK3R1*. This study hereby presents a 15-year-old female with intrauterine growth restriction, short stature, teething delay, characteristic facial gestalts who was identified a novel de novo nonsense mutation in *PIK3R1.*

**Case presentation:**

The proband was admitted to our department due to irregular menstrual cycle and hirsutism with short stature, who had a history of intrauterine growth restriction and presented with short stature, teething delay, characteristic facial gestalts, hirsutism, and thyroid disease. Whole-exome sequencing and Sanger sequencing revealed c.1960C > T, a novel de novo nonsense mutation, leading to the termination of protein translation (p. Gln654*).

**Conclusions:**

This is the first case report of SHORT syndrome complicated with thyroid disease in China, identifying a novel de novo heterozygous nonsense mutation in *PIK3R1* gene (p. Gln654*). The phenotypes are mildly different from other cases previously described in the literature, in which our patient presents with lipoatrophy, facial feature, and first reported thyroid disease. Thyroid disease may be a new clinical symptom of patients with SHORT syndrome.

**Supplementary information:**

**Supplementary information** accompanies this paper at 10.1186/s12881-020-01146-3.

## Background

SHORT syndrome (MIM 269880; ORPHA:3163) is a rare genetic disease whose name is given by Gorlin [1] with the acronyms of short stature (S), hyperextensibility of joints (H), ocular depression (O), Rieger anomaly (R) and teething delay (T) [2, 3]. A few cases have been reported in the literature, but the prevalence of SHORT syndrome remains unclear (< 1:1000000) [4].

The phenotypic presentation most frequently observed in SHORT syndrome are mild intrauterine growth restriction (IUGR), short stature, and a characteristic facial gestalt (e.g. triangular face with a small thin, thin lip, downturned mouth, low-set posteriorly rotated ears, prominent forehead, underdeveloped or thin nasal alae as well as wrinkles) [3–6]. Lipodystrophy, characterized by selective loss of adipose tissue, is another typical feature of the syndrome. It is displayed mainly in the face, chest and upper extremities, often sparing the buttocks and legs [5, 7], causing an aged appearance of the patients suffering from the SHORT syndrome. Insulin resistance is another common characteristic of the disease while ages at diagnosis is highly variable [4, 8]. Severe insulin resistance may also lead to an early onset of type 2 diabetes, typically occurring in the second decade of life [9]. Additionally, almost all postpubertal women affected present polycystic ovary syndrome [4, 10]. There are some other common features of the SHORT syndrome, e.g. ophthalmic abnormalities such as Rieger anomaly, ocular anterior chamber dysgenesis, higher ocular pressure and glaucoma; dental abnormalities such as delayed teething, small teeth and decreased number of teeth; skeletal abnormalities such as delayed bone age, hyperextensibility of the joints as well as clinodactyly [7, 11, 12]. Other less common manifestations include delayed speech development, sensorineural hearing loss, congenital heart defects as well as mild cognitive delay [4, 13]. Intelligence is within normal range and most patients can have normal educational achievements [4].

*PIK3R1* gene encodes the regulatory subunit of the phosphoinositide 3-kinase (PI3K) holoenzyme, activating the AKT/mammalian target of rapamycin (mTOR) pathway to modulate cell proliferation and growth [3]. It is located at 5q13.1 region, containing 16 exons and encoding 724 amino acids. *PIK3R1* mutation is associated with two different conditions, that are SHORT syndrome and a rare primary immunodeficiency disorder named Activated PI3K-delta Syndrome 2 (APDS2, MIM615513) [14].

Here, we report a SHORT syndrome case of a 15-year old female patient in China, exhibiting the classical features of a characteristic facial gestalt, IUGR and delayed teething, though without polycystic ovary syndrome. The patient also presented diffuse thyroid disease, which hasn’t been reported in previous studies. Whole exome sequencing (WES) identified a novel de novo heterozygous mutation(c.1960C > T, p.Gln654*) of the *PIK3R1* gene.

## Case presentation

The patient was a girl born to a physically healthy and non-consanguineous couple by spontaneous delivery at the 37th week. Birth weight was 2150 g (− 3.39SD) and birth length was 44 cm (− 3.41SD), indicating that the patient had intrauterine growth restriction (IUGR). The proband also had teething delay, getting the first tooth at 1 year old. During childhood, the patient was bothered by short stature. Psychomotor and speech development was normal. The height of proband’s father and mother was 168 cm and 155 cm respectively. The patient also had a healthy 20-month-old brother.

**Fig. 1:**
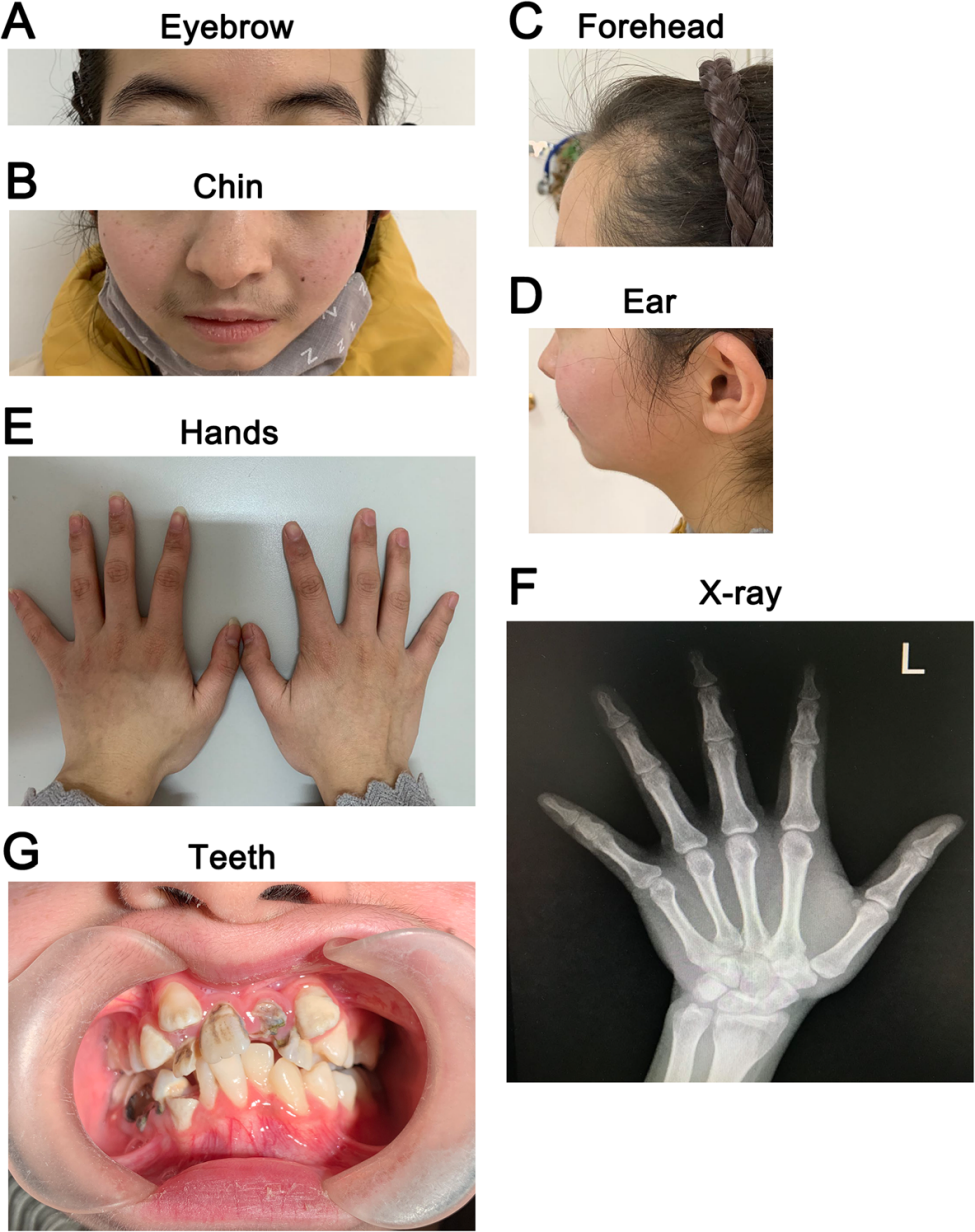
Image of the patient. **a**, **b**, **c**, **d**. Facial abnormalities included triangular face with a small chin, thin lip, downturned mouth, low-set posteriorly rotated ears, obvious beard and bushy eyebrows. **e**, **f**. Thicken and short second phalanx of little finger in the left hand. **g**. Overcrowded and irregular teeth, multiple dental caries

At the age of 15 years and 4 months, the proband was referred to our department due to irregular menstrual cycle and hirsutism with a height of 149 cm (− 2.04SD), weight of 43 kg (− 1.22SD) and body mass index (BMI) of 19.4 kg/m^2^. The height of the proband had remained 149 cm, ever since 13 years old. Physical examination showed a triangular-shaped face, small chin, large low-set ears, thin lip, downturned mouth, obvious beard and bushy eyebrows (Fig. 1a,b,c,d). Oral examination showed overcrowded and irregular teeth, hypodontia, and severe dental caries (Fig. 1g). Pubertal development was assessed according to the Tanner stage, with pubic hair at PH5 stage and breast at B2 stage. The second phalanx of little finger in the left hand was short and thicken, which was confirmed with X-ray (Fig. 1e,f). Ultrasound of neck showed diffuse thyroid disease. Ultrasound biomicroscopy of the eyes, examination of ocular fundus, abdominal ultrasound, reproductive system ultrasound, and chest X-ray were normal. The cranial magnetic resonance imaging (MRI) indicated a small posterior pituitary.

Laboratory investigations revealed normal levels of routine blood test, liver function, insulin-like growth factor-1 (IGF-1, 424 ng/ml), dehydroepiandrosterone sulfate, 17-hydroxyprogesterone, cortisol, adrenocorticotropic hormone, β-HCG, follicle-stimulating hormone (FSH), luteinizing hormone (LH), estradiol, free triiodothyronine (FT3), free thyroxine (FT4), thyroid-stimulating hormone (TSH), triglyceride, cholesterol, and low-density lipoprotein. Abnormal laboratory results of the proband were shown in Table 1. Oral glucose tolerance test indicated insulin resistance while blood glucose was normal (Supplementary Table S1).

**Table 1:** Laboratory results of the patient

Factor	Value	Reference range
High-density lipoprotein (mmol/L)	0.75	0.90–1.68
Apolipoprotein A1(g/L)	0.93	1.04–2.02
Apolipoprotein B(g/L)	0.4	0.66–1.33
Androstenedione (ng/ml)	5.59	0.10–2.99
Testosterone (nmol/L)	3.23	< 1.39
IgA(g/L)	3.22	0.67–3.14
Cytotoxic T cell (cells/ul)	1074.02	200–900
B cell (cells/ul)	522.85	100–500
Thyroidperoxidase antibodies (TPOAb)(IU/ml)	223.7	0–34
Antithyroglobulin antibodies (TGAb)(IU/ml)	219.6	0–115

**Fig. 2:**
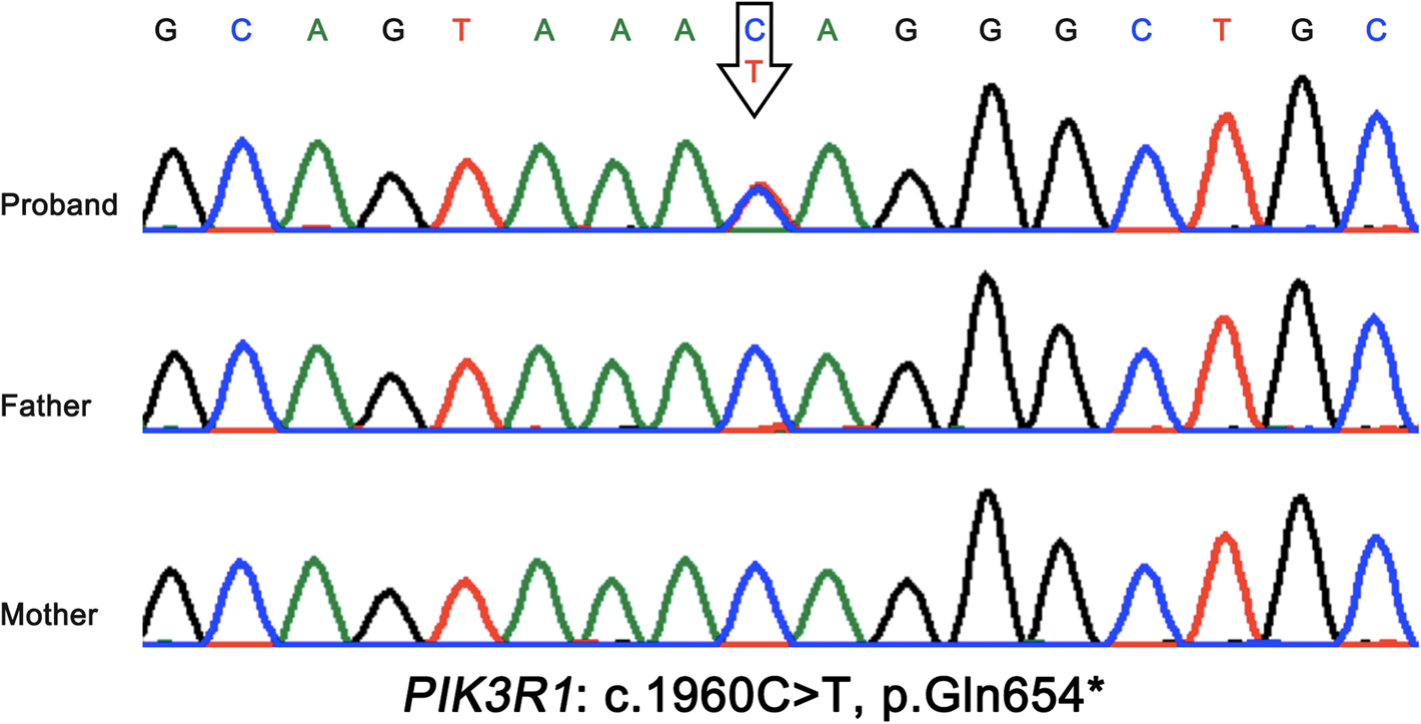
Sanger sequencing showed a heterozygous nonsense mutation (c.1960C > T, p.Gln654X in exon 11) in the patient, and the parents were normal. Black arrows, mutant bases

WES was performed to make a clear clinical diagnose. The candidate variants were first screened by a minor allele frequency < 3% against the 1000 Genomes Project, the NHLBI exome variant server or in 50 HapMap control exomes. Then, short stature, facial abnormalities were selected as the filtering clinical symptoms to analyze the screened candidate variants. According to the guidelines recommended by the American College of Medical Genetics and Genomics, a pathogenic variant of *PIK3R1* gene was identified to contribute to the patient’s conditions. Sequencing result indicated c.1960C > T of *PIK3R1* gene a novel nonsense mutation, leading to the termination of protein translation (p. Gln654*), which was confirmed by sanger sequencing (Fig. 2). In addition, direct sequencing results showed the genotypes of proband’s parents were wild-type, suggesting it was a de novo mutation.

**Fig. 3:**
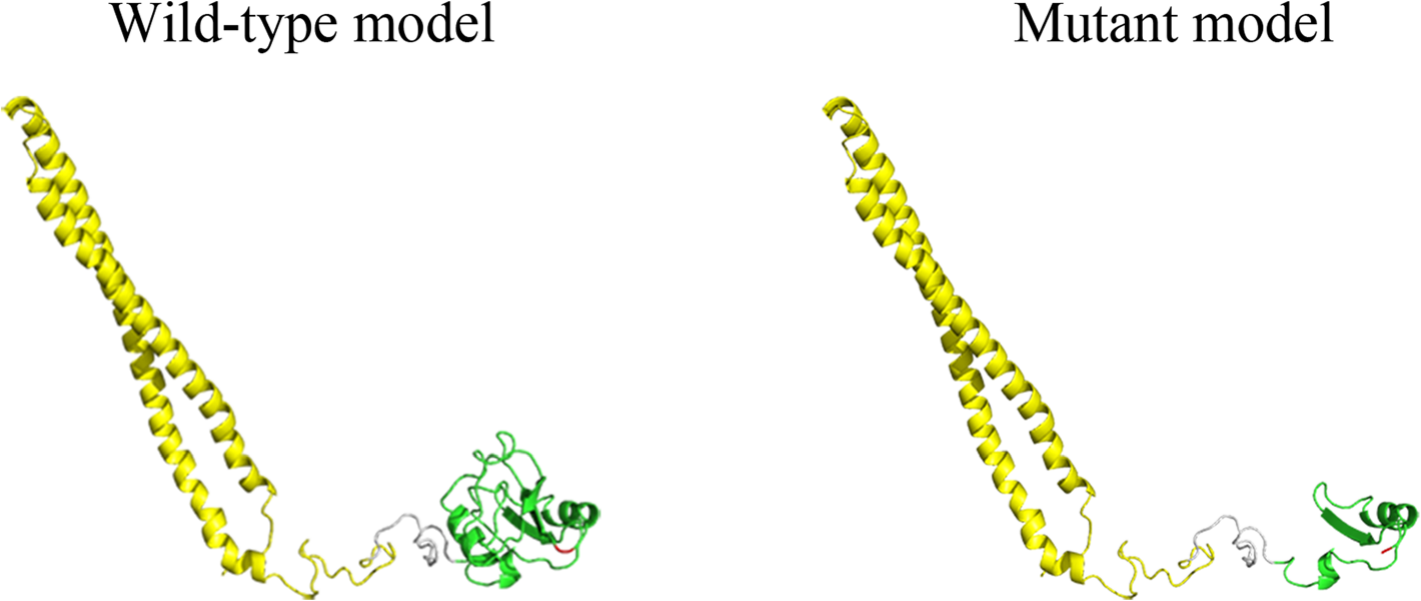
Homology models of the wild-type *PIK3R1* and p.Gln654* mutant *PIK3R1*. The inter src-homology 2(iSH2) domain and C-terminal src-homology (cSH2) domain are shown in yellow and green respectively, with the *PIK3R1* G654 shown in red

The three-dimensional structure of the wild-type (WT) PIK3R1 protein was generated by the SWISS-MODEL online server and was examined using Pymol v.1.8.4.0 software (https://www.pymol.org; Schrödinger, New York, NY, USA). The three-dimensional structure of the mutant PIK3R1 protein was generated by deleting the amino acid after glutamic acid 654. GMQE (Global Model Quality Estimation) and QMEAN for the model are 0.24 and − 1.83, indicating the model is in good quality. The model shows that *PIK3R1* G654 is located on the loop in the C-terminal src-homology 2 (cSH2) domain (Fig. 3). The termination of protein translation leading to the damage of the cSH2 domain (Fig. 3).

## Discussion and conclusion

SHORT syndrome is inherited in an autosomal dominant manner confirmed by the identification of heterozygous mutations in *PIK3R1* (phosphoinositide-3-kinase regulatory subunit 1, MIM 171833) [15], except for two cases that were resulted from the mutation of *PRKCE* [16] and *IGF1R* [17] respectively. To date, 12 variants have been proven to be SHORT syndrome and the missense mutation of c.1945C > T (p. Arg649Trp) was the most common variant [7, 13]. The mutants are distributed in exons 12, 13, 15, 16, and intron 11 (Supplementary Fig.S1). Exon 15, encoding the cSH2 domain, covers most of the mutations (8/12), including all nonsense mutations (p.Lys653*, p.Gln654* (in this study), p.Tyr657*). Apparently, exon 15 is a mutational hot spot.

The proband presented with some common phenotypes such us IUGR (28/32), short stature (33/41), teething delay (26/27), characteristic facial dysmorphim (43/43), and insulin resistant (14/22) (Table 2). However, it should be noted that some common clinical symptoms aren’t observed on the patient, e.g., ocular depression (35/37) and lipoatrophy (34/41). As almost all previous cases are from North America and Europe, natural physiological differences between races might be the differentiating factor. The clinical manifestations of SHORT syndrome in patients with *PIK3R1* mutation in different exons/introns may facilitate the understanding of genotype-phenotype correlations (Table 2). All patients with SHORT syndrome have characteristic facial gestalts, making it an essential symptom (43/43). Not all features described in the acronyms of SHORT syndrome are universally seen. A 32 cases meta-analysis showed only half of the patients have four or more of classic features [3, 4], which is consistent with our findings. Because of lack of cases in different domains, we could not illustrate specific genotype-phenotype correlations. We also analyzed the phenotypes of cases carrying nonsense mutations comparing with other cases, it seems that nonsense mutations have no correlation with severer or specific phenotypes, probably because the mutations are in the second to last exon, having limited influence on the protein (Supplementary Table S2).

**Table 2:** Clinical Features of the patient and reported SHORT patients with Mutations in *PIK3R1*

	The proband	Domain					
		na	iSH2		cSH2		na
Mutation site	Exon 15	Intron 11	Exon 12	Exon 13	Exon 15	Exon 16	Total
Number of cases	1	3	1	1	36	2	43
Sex	F	F1/M2	F	M	F21/M15	M2	F23/M20
Premature birth	–	0/2	1/1	1/1	5/22	nd	7/26
IUGR	+	nd	1/1	1/1	26/28	0/2	28/32
Weight at birth <3rd per	+	2/2	1/1	1/1	25/27	0/2	29/33
OFC at birth <3rd per	nd	0/1	1/1	1/1	9/13	nd	11/16
SHORT acronym signs							
Short stature	+	1/3	1/1	1/1	30/34	0/2	33/41
Hyperextensibility of joints	–	1/2	0/1	0/1	7/25	0/2	8/31
Ocular depression	–	1/1	1/1	1/1	30/32	2/2	35/37
Rieger anomaly	–	nd	0/1	0/1	14/33	2/2	16/37
Teething delay	+	2/2	1/1	1/1	21/22	1/1	26/27
Other signs							
Characteristic facial dysmorphim	+	3/3	1/1	1/1	36/36	2/2	43/43
Progeroid appearance	+	2/2	nd	nd	14/14	nd	16/16
Lipoatrophy	–	2/2	1/1	1/1	30/35	0/2	34/41
Thin, wrinkled skin with readily visible veins	nd	1/1	1/1	1/1	15/20	0/2	18/25
Ophthalmological abnormalities							
Glaucoma	–	nd	nd	nd	3/13	nd	3/13
Hyperopia	–	nd	1/1	1/1	5/10	0/2	7/14
Astigmatism	–	nd	0/1	1/1	4/12	nd	5/14
Myopia	+	nd	0/1	0/1	5/13	nd	5/15
Overcrowded teeth	+	1/1	nd	nd	4/5	nd	5/6
Delayed bone age	–	nd	1/1	1/1	4/8	nd	6/10
Inguinal hernia	nd	nd	0/1	0/1	3/19	nd	3/21
Intellectual deficiency	–	1/3	nd	nd	2/17	0/2	3/22
Speech delay	–	1/1	1/1	1/1	10/18	1/2	14/23
Diabetes	–	nd	0/1	0/1	10/21	nd	10/23
Insulin resistance	+	nd	1/1	1/1	11/20	nd	14/22
Hearing loss	–	3/3	nd	nd	5/13	1/2	9/18
Frequent infections^a^	–	3/3	0/1	0/1	3/8	nd	6/13
Congenital heart diseases^b^	–	2/2	nd	nd	1/2	nd	3/4
Pulmonary stenosis^c^	–	1/1	nd	nd	3/4	nd	4/5
Ovarian cysts^d^	–	nd	nd	na	8/9	na	8/9

*IUGR* intrauterine growth restriction; occipitofrontal circumference; *SHORT* short stature (S), hyperextensibility of joints (H), ocular depression (O), Rieger abnormality (R) and teething delay (T); *na* not applicable; and *nd* no data

^a^ Frequent infections include respiratory infection, pneumonia, and urinary infection

^b^ Congenital heart diseases include mitral dysplasia, and ventricular septal defect

^c^ This contains a case of pulmonary hypertension

^d^ This contains a case of Ovarian cancer

We describe the dental status of the patient with SHORT syndrome in detail. According to our review, teething delay is common (26/27) but a detailed observation of dental status is often ignored by researchers. Usually, inherence plays an important role in teeth development. Thus, dental problems like overcrowded teeth (5/6), hypodontia, and dental caries in SHORT syndrome patients warrant a closer look and early identification before they progress further down the road.

Diffuse thyroid disease with positive TPOAb and TGAb has not been reported before in patients with SHORT syndrome [4]. *PIK3R1* RNA is expressed moderately in the thyroid and it regulates PI3K–AKT signaling pathway [3]. Evidence shows that PI3K–AKT signaling pathway is important in the progression of thyroid cancer, it is touted to be a target of a possible treatment for patients with advanced types of thyroid carcinoma [18]. On the other hand, the relationship between insulin resistance and thyroid function have been controversial [19]. Positive TPOAb and TGAb do exist in the general population. In this patient, we could not make sure whether it is induced by the mutation directly or not. So far, the patient’s thyroid function seems to be normal. Still, a future follow-up is needed as thyroid disease may be a new clinical symptom of patients with SHORT syndrome and more evidence is necessary to support this hypothesis.

As mentioned above, APDS2 can be triggered by *PIK3R1* mutations. So, it is important to distinguish APDS2 from SHORT syndrome. Features of APDS2 are recurrent upper tract respiratory infections and lymphoproliferation. Immunological evaluation of the patient may show elevated IgM, IgA deficiency, low CD4 and CD8 naïve T cell counts, and B-cell lymphopenia. It should be noted that 3 cases have been reported with diagnosis of SHORT syndrome and APDS2 synchronously [14, 20]. The 3 cases are all splicing variants, resulting in the skipping of exon 11 [14]. Exon 11 encodes the inter-SH2 domain of the p85α isoform, associating to the p110δ catalytic subunit binding [21]. All of the studies above have different findings compare to that of our patient’s mildly abnormal level of immune cells and elevated IgA level. Besides, our patient did not have a history of recurrent infection, which indicates there was no serious problem with her immune system. Thus, the diagnosis of APDS2 can be excluded from our patient. It is generally recommended that APDS2 patients be assessed by a clinical geneticist to exclude concurrence with SHORT syndrome. Conversely, patients with SHORT syndrome should have their immune system assessed, especially when they have a history of recurrent respiratory infections and/or lymphoproliferation [14].

Polycystic ovary syndrome (PCOS) develops almost in all postpubertal women (8/9) with SHORT syndrome [4], whose features include irregular menstrual cycle, hirsutism, acne, hyperandrogenemia, and insulin resistance. Elevated androstenedione and testosterone level could explain the hirsutism and irregular menstrual cycle of our patient. However, normal reproductive system ultrasound did not support the diagnosis of PCOS. Still, considering the more stringent diagnostic criteria for PCOS in adolescence compared to that of adults, there is a considerable chance for our patient to be diagnosed with PCOS later in her adult life.

In conclusion, our study reported on a Chinese patient with SHORT syndrome, identifying a novel de novo heterozygous nonsense mutation in the *PIK3R1* gene (p. Gln654*). The phenotypes of the proband were mildly different from other cases previously reported. Common clinical symptoms like ocular depression and lipoatrophy weren’t observed on the patient while diffuse thyroid disease was presented. We aimed to expand the spectrum of genotypes and phenotypes of SHORT syndrome through this case report. Nevertheless, the precise genotype-phenotype correlations and molecular mechanisms of SHORT syndrome remain elusive and further study is warranted.

## Supplementary information

**Additional file 1: Table S1.** Oral glucose tolerance test of the patient.

**Additional file 2: Table S2.** Clinical Features of the patients with nonsense mutations in *PIK3R1.*

**Additional file 3: Figure S1.** The schematic diagram of the distribution of 12 reported mutations as well as p.Gln654* in *PIK3R1* gene.

## Data Availability

The raw datasets generated and/or analysed during the current study are not publicly available in order to protect participant confidentiality. The data and materials are available from the corresponding author upon reasonable request.

## Abbreviations

IUGR: Intrauterine growth restriction
PI3K: Phosphoinositide 3-kinase
WES: Whole exome sequencing
MRI: Magnetic resonance imaging
PCOS: Polycystic ovary syndrome

## References

[1] Gorlin RJ, Cervenka J, Moller K, Horrobin M, Witkop CJ (1975). Malformation syndromes. A selected miscellany. Birth Defects Orig Artic Ser.

[2] Lipson AH, Cowell C, Gorlin RJ (1989). The SHORT syndrome: further delineation and natural history. J Med Genet.

[3] Innes AM, Adam MP, Dyment DA, SHORT Syndrome, in GeneReviews(®) (1993). University of Washington, Seattle copyright © 1993-2020, University of Washington, Seattle. GeneReviews is a registered trademark of the University of Washington, Seattle.

[4] Avila M, Dyment DA, Sagen JV, St-Onge J, Moog U, Chung BHY (2016). Clinical reappraisal of SHORT syndrome with PIK3R1 mutations: toward recommendation for molecular testing and management. Clin Genet.

[5] Chudasama KK, Winnay J, Johansson S, Claudi T, König R, Haldorsen I (2013). SHORT syndrome with partial lipodystrophy due to impaired phosphatidylinositol 3 kinase signaling. Am J Hum Genet.

[6] Dyment DA, Smith AC, Alcantara D, Schwartzentruber JA, Basel-Vanagaite L, Curry CJ (2013). Mutations in PIK3R1 cause SHORT syndrome. Am J Hum Genet.

[7] Klatka M, Rysz I, Kozyra K, Polak A, Kołłątaj W (2017). SHORT syndrome in a two-year-old girl - case report. Ital J Pediatr.

[8] Huang-Doran I, Tomlinson P, Payne F, Gast A, Sleigh A, Bottomley W (2016). Insulin resistance uncoupled from dyslipidemia due to C-terminal PIK3R1 mutations. JCI Insight.

[9] Thauvin-Robinet C, Auclair M, Duplomb L, Caron-Debarle M, Avila M, St-Onge J (2013). PIK3R1 mutations cause syndromic insulin resistance with lipoatrophy. Am J Hum Genet.

[10] Lewandowski KC, Dąbrowska K, Brzozowska M, Kawalec J, Lewiński A (2019). Metformin paradoxically worsens insulin resistance in SHORT syndrome. Diabetol Metab Syndr.

[11] Bárcena C, Quesada V, De Sandre-Giovannoli A, Puente DA, Fernández-Toral J, Sigaudy S (2014). Exome sequencing identifies a novel mutation in PIK3R1 as the cause of SHORT syndrome. BMC Med Genet.

[12] Brodsky MC, Whiteside-Michel J, Merin LM (1996). Rieger anomaly and congenital glaucoma in the SHORT syndrome. Arch Ophthalmol.

[13] Schroeder C, Riess A, Bonin M, Bauer P, Riess O, Döbler-Neumann M (2014). PIK3R1 mutations in SHORT syndrome. Clin Genet.

[14] Bravo García-Morato M, García-Miñaúr S, Molina Garicano J, Santos Simarro F, Del Pino ML, López-Granados E (2017). Mutations in PIK3R1 can lead to APDS2, SHORT syndrome or a combination of the two. Clin Immunol.

[15] Chung BK, Gibson WT (2014). Autosomal dominant PIK3R1 mutations cause SHORT syndrome. Clin Genet.

[16] Alcantara D, Elmslie F, Tetreault M, Bareke E, Hartley T, Majewski J (2017). SHORT syndrome due to a novel de novo mutation in PRKCE (protein kinase Cɛ) impairing TORC2-dependent AKT activation. Hum Mol Genet.

[17] Prontera P, Micale L, Verrotti A, Napolioni V, Stangoni G, Merla G (2015). A new homozygous IGF1R variant defines a clinically recognizable incomplete dominant form of SHORT Syndrome. Hum Mutat.

[18] Nozhat Z, Hedayati M (2016). PI3K/AKT pathway and its mediators in thyroid carcinomas. Mol Diagn Ther.

[19] Amouzegar A, Kazemian E, Gharibzadeh S, Mehran L, Tohidi M, Azizi F (2015). Association between thyroid hormones, thyroid antibodies and insulin resistance in euthyroid individuals: a population-based cohort. Diabetes Metab.

[20] Petrovski S, Parrott RE, Roberts JL, Huang H, Yang J, Gorentla B (2016). Dominant splice site mutations in PIK3R1 cause hyper IgM Syndrome, lymphadenopathy and short stature. J Clin Immunol.

[21] Lucas CL, Chandra A, Nejentsev S, Condliffe AM, Okkenhaug K (2016). PI3Kδ and primary immunodeficiencies. Nat Rev Immunol.

