# Supplementary material for: A novel PIK3R1 mutation of SHORT syndrome in a Chinese female with diffuse thyroid disease: a case report and review of literature
Source: BMC Med Genet. 2020 Oct 31;21:215. doi: 10.1186/s12881-020-01146-3 (PMC7603772; doi:10.1186/s12881-020-01146-3)
Supplement: Supplementary file 1 — Additional file 1: Table S1. Oral glucose tolerance test of the patient. [file 12881_2020_1146_MOESM1_ESM.docx]

Supporting Information Table S1. Oral glucose tolerance test of the patient

| Time | Blood glucose | Insulin | Serum C peptide |
| --- | --- | --- | --- |
| 0min | 5.18 | 135.4 | 1020 |
| 30min | 8.05 | 2224.5 | 6090 |
| 60min | 6.28 | 2708.6 | 7770 |
| 120min | 6.12 | 1518.9 | 5520 |
| 180min | 5.41 | 516 | 3450 |
| reference range | 3.6-6.11mmol/L | 17.8-173pmol/L | 370-1470pmol/L |
