# Supplementary material for: A novel PIK3R1 mutation of SHORT syndrome in a Chinese female with diffuse thyroid disease: a case report and review of literature
Source: BMC Med Genet. 2020 Oct 31;21:215. doi: 10.1186/s12881-020-01146-3 (PMC7603772; doi:10.1186/s12881-020-01146-3)
Supplement: Supplementary file 2 — Additional file 2: Table S2. Clinical Features of the patients with nonsense mutations in PIK3R1. [file 12881_2020_1146_MOESM2_ESM.docx]

Supporting Information Table S2. Clinical Features of the patients with nonsense mutations in *PIK3R1*

| Mutation | p.Gln654* | p.Lys653* | p.Tyr657∗ | Total |
| --- | --- | --- | --- | --- |
| cases | 1 | 1 | 2 | 4 |
| Sex | F | F | F1/M1 | F3/M1 |
| Premature birth | 0/1 | 0/1 | 0/2 | 0/4 |
| IUGR | 1/1 | 1/1 | 2/2 | 4/4 |
| Weight at birth < 3^rd^ per | 1/1 | 1/1 | 2/2 | 4/4 |
| OFC at birth < 3^rd^ per | nd | 1/1 | 0/1 | 1/2 |
| SHORT acronym signs |  |  |  |  |
| Short stature | 1/1 | 1/1 | 2/2 | 4/4 |
| Hyperextensibility of joints | 0/1 | 0/1 | 1/2 | 1/4 |
| Ocular depression | 0/1 | 1/1 | 2/2 | 3/4 |
| Rieger anomaly | 0/1 | 0/1 | 0/2 | 0/4 |
| Teething delay | 1/1 | 1/1 | 1/1 | 3/3 |
| Other signs |  |  |  |  |
| Characteristic facial dysmorphim | 1/1 | 1/1 | 2/2 | 4/4 |
| Progeroid appearance | 1/1 | nd | 1/1 | 2/2 |
| Lipoatrophy | 0/1 | 1/1 | 1/2 | 2/4 |
| Thin, wrinkled skin with readily visible veins | nd | 1/1 | nd | 1/1 |
| Ophthalmological abnormalities |  |  |  |  |
| Glaucoma | 0/1 | 0/1 | 0/1 | 0/3 |
| Hyperopia | 0/1 | nd | nd | 0/1 |
| Astigmatism | 0/1 | nd | nd | 0/1 |
| Myopia | 1/1 | 0/1 | nd | 1/2 |
| Overcrowded teeth | 1/1 | nd | 1/1 | 2/2 |
| Delayed bone age | 0/1 | 1/1 | 1/1 | 2/3 |
| Inguinal hernia | nd | nd | 0/1 | 0/1 |
| Intellectual deficiency | 0/1 | 0/1 | nd | 0/2 |
| Speech delay | 0/1 | 1/1 | 1/1 | 2/3 |
| Diabetes | 0/1 | 0/1 | 1/2 | 1/4 |
| Insulin resistance | 1/1 | 0/1 | 1/2 | 2/4 |
| Hearing loss | 0/1 | 1/1 | nd | 1/2 |
| Frequent infections^a^ | 0/1 | nd | 0/1 | 0/2 |
| Congenital heart diseases^b^ | 0/1 | nd | nd | 0/1 |
| Pulmonary stenosis^c^ | 0/1 | nd | nd | 0/1 |
| Ovarian cysts^d^ | 0/1 | nd | 1/1 | 1/2 |

IUGR, intrauterine growth restriction; occipitofrontal circumference; SHORT, short stature (S), hyperextensibility of joints (H), ocular depression (O), Rieger abnormality (R) and teething delay (T); na, not applicable; and nd, no data.

^a^ Frequent infections include respiratory infection, pneumonia, and urinary infection.

^b^ Congenital heart diseases include mitral dysplasia, and ventricular septal defect.

^c^ This contains a case of pulmonary hypertension.

^d^ This contains a case of Ovarian cancer.
