# Supplementary material for: A novel PIK3R1 mutation of SHORT syndrome in a Chinese female with diffuse thyroid disease: a case report and review of literature
Source: BMC Med Genet. 2020 Oct 31;21:215. doi: 10.1186/s12881-020-01146-3 (PMC7603772; doi:10.1186/s12881-020-01146-3)
Supplement: Supplementary file 3 — Additional file 3: Figure S1. The schematic diagram of the distribution of 12 reported mutations as well as p.Gln654* in PIK3R1 gene. [file 12881_2020_1146_MOESM3_ESM.docx]

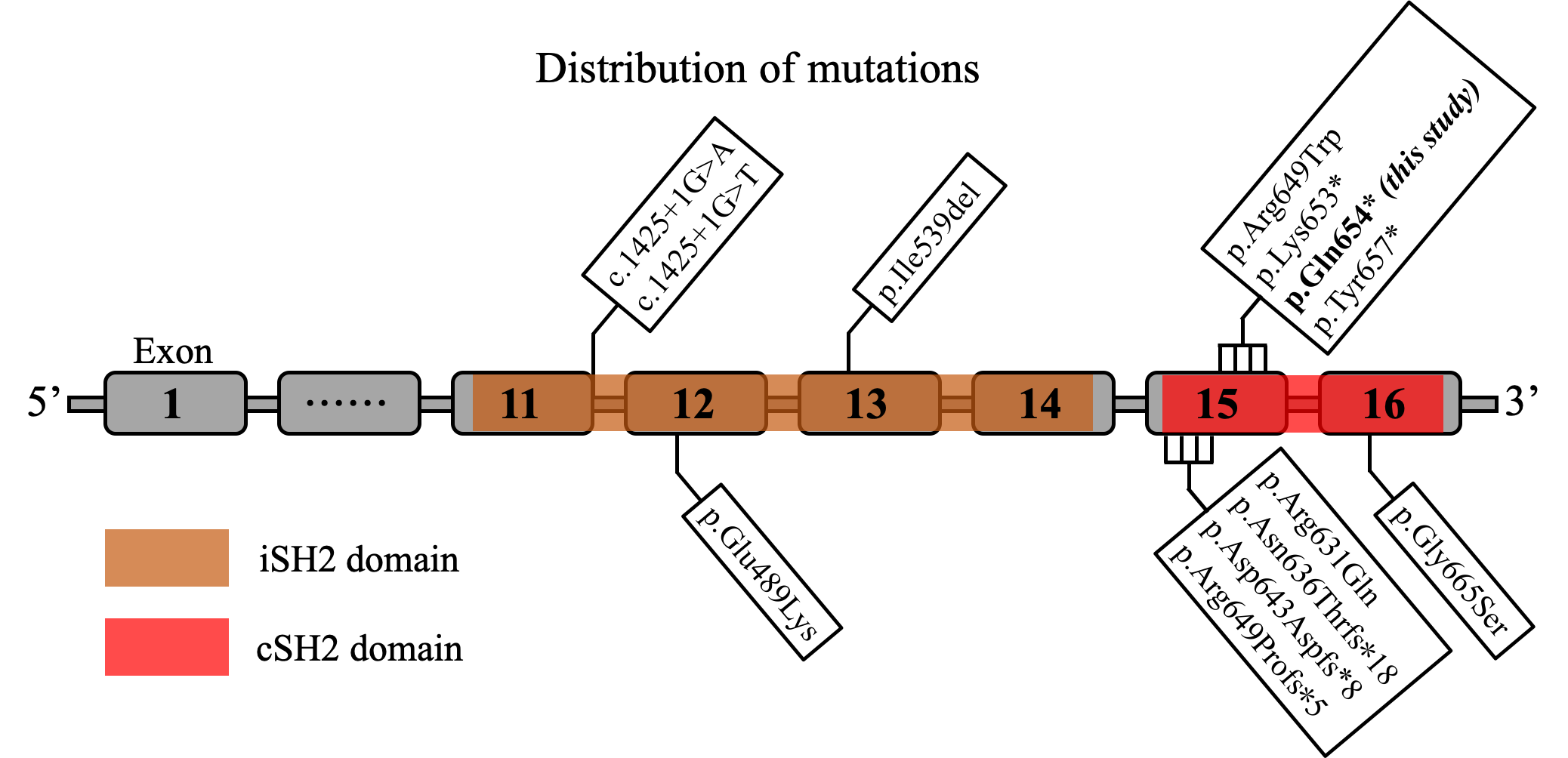


Supplementary Figure S1. The schematic diagram of the distribution of 12 reported mutations as well as p.Gln654* in *PIK3R1* gene.
